# Tailoring visible-light active TiO_2_/cellulose nanocomposites with controlled crystalline structure for enhanced photocatalytic performance

**DOI:** 10.1038/s41598-023-50749-2

**Published:** 2024-01-02

**Authors:** Nutsupa Pimsawat, Somnuk Theerakulpisut, Khanita Kamwilaisak

**Affiliations:** 1 Department of Chemical Engineering, Faculty of Engineering, Khon Kaen University, Khon Kaen, 40002 Thailand; 2 Energy Management and Conservation Office, Faculty of Engineering, Khon Kaen University, Khon Kaen, 40002 Thailand

**Keywords:** Chemistry, Materials science

## Abstract

This work involves a green and simple synthesis of TiO_2_ nanoparticles on cellulose under mild conditions without the need for calcination via hydrolysis of titanium oxysulfate (TiOSO_4_). The synthesis conditions, such as sulfuric acid concentration (0–10% wt), temperature (70–90 ℃), and time (4–8 h), focused on precisely controlling the structure of TiO_2_ to enhance its photocatalytic effectiveness under visible light. At a lower 2.5 wt% sulfuric acid concentration, pure anatase was formed on the cellulose, while an increase in the range of 5.0–7.5 wt% sulfuric acid concentration yielded a rutile phase, resulting in a mixed phase of anatase and rutile on the cellulose. The pure rutile phase was found at a low temperature (70 ℃), while increased temperature led to the formation of the anatase phase. These results confirmed that the formation of crystalline TiO_2_ phase on the cellulose depended on sulfuric acid concentration and temperature for hydrolysis. Additionally, the photocatalytic properties of the obtained materials were evaluated by degradationvisible of Rhodamine B (RhB) under UV and visible light. The findings revealed that the mixed phase (anatase/rutile) of TiO_2_ on the cellulose demonstrated a superior photocatalytic efficiency (99.2%) compared to pure anatase (85.75%) and rutile (75.08%) when exposed to visible light.

## Introduction

Semiconductors are highly useful materials with high potential for many industrial applications such as energy and photocatalysis^1^. Photocatalysis of semiconductor can be used in the production of chemicals by degradation of biomass, and removal of pollution such as microplastic^2^ and organic contamination^3^ for water treatment. Among the semiconductors used for photocatalysis, titanium dioxide (TiO_2_) has outstanding properties such as environmental friendliness, low cost, thermal and chemical stability and superior photocatalytic activity^1,3–7^. The photocatalytic efficiency of TiO_2_ depends on its crystalline polymorph, morphology, particle size, and surface area^6,8^.

In general, TiO_2_ are available in three most commonly phases: anatase, rutile and brookite. Anatase and rutile phases have been reported in the literature more than brookite due to it’s simplicity in synthesis. Moreover, anatase phase is the most reported for photocatalysis than rutile due to rutile having lower energy band gap causing inhibition of photocatalytic activity from fast recombination between the electrons and the holes. In addition, it is reported that a mixed-phase between anatase and rutile promotes greater photocatalytic activity more than only anatase phase, owing to efficient electron–hole separation from interconnection between anatase and rutile^3,9,10^. The best ratio between anatase and rutile for photocatalytic activity has been reported at 80:20^3,11^ or 70:30^10^, consistent with the ratio of mixed-phase TiO_2_ in commercial grade, called Degussa P25, which is most used as a standard for evaluating photocatalytic activity of other photocatalysts. Therefore, synthesis of mixed-phase TiO_2_ has received a great deal of research attention. The different phase ratio of TiO_2_ depends on the method and condition for synthesis, especially temperature for calcination^6^. Normally, anatase phase was found in the initial period of synthesis after which the rutile phase was formed after an increase in calcination temperature (> 500 ℃), and eventually formation of only rutile phase was found (> 800 ℃)^12^. However, agglomeration of TiO_2_ nanoparticles during the synthesis, storage, transportation and use was found to cause to a decrease in photocatalytic efficiency. Moreover, due to the small particle size, recovery of the TiO_2_ nanoparticles for reuse was difficult^4,5,7,13^. Consequently, immobilized particles on support were generally used in most studies to prevent the agglomeration.

Various materials such as biomass^14^, polymer^15^, glass fiber^16^, wood^17^ and cellulose^18^ were used as supporting material of nanoparticles. Among supporting materials, cellulose is widely used due to its abundance, low cost and high hydroxyl content. Some research reported that hydroxyl groups on cellulose surface can act as a template to accelerate nucleation and growth of TiO_2_ nanoparticles^7^. Moreover, cellulose can increase the absorption wavelength, resulting in an increase of photocatalytic efficiency. TiO_2_ on cellulose was used in various applications such as adsorption, antibacterial activity^19^, photodegradation^4^, cosmetic^20^ and energy storage^21^. Normally, the crystalline phase of TiO_2_ was formed after calcination at higher than 400 ℃, but the temperature would degrade the cellulose support. Therefore, the process at low temperature for synthesis of TiO_2_ nanoparticles was selected in this study.

A number of methods for synthesis of TiO_2_ on cellulose support were reported such as dip coating^22^, mixing^23^, hydrothermal process^24^, microwave irradiation^13^ and hydrolysis^4,5,7,20,25^. Mixing is a simple method; however, it exhibited the lower interaction between cellulose and nanoparticles while the hydrothermal method required high temperature and specific equipment. Microwave irradiation has been used to reduce the reaction time but at a high cost of equipment. Among the processes, hydrolysis is considered attractive due to low temperature and requires no chemical agent. The popular precursor used for synthesis of TiO_2_ on cellulose is titanium alkoxide such as titanium oxysulfate (TiOSO_4_)^4,7,13,20^, titanium (IV) isopropoxide (TTIP)^26^ and titanium *n*-butoxide^27^. Among the alkoxide, TiOSO_4_ is the most attractive due to low cost and ease of handling^7,28^.

The factors which influence synthesis of TiO_2_ by the hydrolysis process include temperature, acid concentration, precursor concentration and time. The temperature used by most researchers was between 70 and 110 ℃. The formation of crystalline phase of TiO_2_ on cellulose was found to be pure anatase^4,25^ and pure rutile^7^, depending on temperature. Acid type also affects the formation of crystalline phase. Sulfuric acid (H_2_SO_4_) yields the anatase phase whereas hydrochloric acid (HCl) produces the rutile phase. The mixed phase can be obtained when nitric acid (HNO_3_) is used^29,30^. However, pure anatase and rutile phase of TiO_2_ with different H_2_SO_4_ concentration were reported by Shandilya and Capron. They found that at a lower concentration (0–0.03 M), no crystalline phase was formed while, between 0.03 and 0.15 M, the anatase phase of TiO_2_ was generated, and between 0.15 and 0.4 M the rutile phase was formed^20^. Moreover, the mixed phase of TiO_2_ on membrane from TTIP via low temperature dissolution precipitation and varying concentration of HCl, temperature and time was reported^10^. However, there has not been any report on the formation of mixed phase TiO_2_ by hydrolysis of TiOSO_4_ under sulfuric acid solution on cellulose in the literature.

In the present work, we report a facile method to synthesize TiO_2_ on cellulose at low temperature and we obtained pure anatase, pure rutile and mixed phase of TiO_2_ without post treatment. This method owns a number of advantages. The process is simple without hazardous chemicals, and low energy is consumed. The photocatalysis of as-synthesized TiO_2_ for degradation of rhodamine B under UV and visible light was investigated. TiO_2_ on cellulose also showed high efficiency for reusability. Moreover, the mechanism of rhodamine B degradation by the synthesized TiO_2_ as a catalyst was studied in detail.

## Materials and methods

### Materials

*Eucalyptus* pulp was obtained from Phoenix Pulp and Paper (Public Company, Thailand). Titanium (IV) oxysulfate (TiOSO_4_) and Rhodamine B were purchased from Sigma-Aldrich (Singapore). Analytical grade sulfuric acid (H_2_SO_4_, 96%), propan-2-ol ((CH_3_)_2_CHOH, 99.8%) and silver nitrate (AgNO_3_, 99.8%) were supplied by R.C.I. Labscan (Thailand). l-Ascorbic acid was purchased from Fisher scientific (United Kingdom). Oxalic acid 2-hydrate was supplied by KEMAUS (Australia).

### Preparation of TiO_2_ on cellulose

A synthesis method of TiO_2_ on cellulose substrate was modified from the method by Shandilya and Capron^20^. In brief, a mixture was prepared by combining 0.25 M of TiOSO_4_ with 40 ml of sulfuric acid aqueous solution. Cellulose was obtained from *Eucalyptus* pulp. The *Eucalyptus* pulp was blended and sieved through an 80-mesh. The sieved pulp (0.5 g) was added to the mixture with continued agitation at a speed of 250 rpm at a reaction temperature of 70 ℃ for a reaction time of 4 h. The solution was then centrifuged and the residue was then washed with DI water until neutralized and then dried in an oven at 40 °C for 24 h. The influence of sulfuric acid aqueous concentration (0–10%), reaction temperature (70, 80 and 90 ℃), and reaction time (4, 6, and 8 h) on the phase structure of TiO_2_ on cellulose was investigated. TiO_2_ without cellulose was synthesized by using 0.25 M of TiOSO_4_ with 40 ml of 5%H_2_SO_4_ at 90 °C for 8 h.

### Characterization

The crystalline structure of all samples was determined by X-ray diffraction (XRD) (Bruker D8 Advance, Germany). The analysis was carried out using the scanning rate of 10°/min and 2θ in the range from 10° to 80°. The crystallinity index (%CrI) of cellulose was calculated following Eq. (1)^31^.

1 $$\%CrI=\frac{{I}_{200}-{I}_{am}}{{I}_{200}}$$

where I_200_ is the maximum intensity of crystallinity plane 200 (2θ = 22.5°), while I_am_ is the intensity of amorphous structure (2θ = 22.5°).

The crystalline size of cellulose and cellulose-TiO_2_ composites was calculated by Scherrer's and the value of the plane spacing (*d*-spacing) was calculated from Bragg’s law^32^

Equation as follows:

2 $$L=\frac{K\lambda }{\beta cos\theta }$$

3 $$d=\frac{\lambda }{2Sin\theta }$$

where L is the crystalline size (nm), *d* is the value of plane spacing (Å), K is a dimensionless shape factor (K = 0.94), λ is the X-ray wavelength (λ = 0.15418 Å), β is half of the maximum intensity (FWHM), and θ is the Bragg angle.

The ratio of rutile phase was calculated by Spurr and Myers’s method^3^:

4 $${\text{W}}_{{\text{R}}} = \frac{1}{{1 + 0.884\frac{{I_{A} }}{{I_{R} }}}}$$

where I_A_ and I_R_ are integrated intensities of anatase (101) and rutile (110) diffraction peak, respectively.

Raman spectroscopy was used to confirmed the crystalline structure of all TiO_2_ on cellulose samples. The spectra were detected by Raman Spectrometer (XploRA plus, Horiba, Kyoto, Japan) with a laser excitation wavelength of 532 nm.

The functional molecule group of all TiO_2_ samples was analyzed by Fourier Transform Infrared Spectrophotometer (FTIR) (Bruker Tensor27 model, Germany). The sample was pressed into pellet form and measured at a resolution of 4 cm^−1^ with an accumulation of 32 scans. The results in the spectra range between 4000 and 500 cm^−1^ were recorded.

The particle size and crystalline structure (Phase) of all samples were detected by Transmission electron microscopy (TEM) and high-resolution TEM (HRTEM) (F.E.I., Model: TECNAI G2 20), respectively.

The morphology of all samples was analyzed using Field Emission Scanning Electron Microscopy (FE-SEM) (F.E.I., Model: Helios NanoLab G3 CX). The amount of elements on the sample was detected using this instrument by EDS.

Thermal stability of all samples was analyzed by thermal gravimetric analysis (DTG 60, Shimadzu, Japan). In brief, the heating rate is 10 °C/min in a temperature range of ambient to 800 °C under a nitrogen gas flow rate of 60 ml/min.

The absorption and reflection of each sample were determined by UV–VIS–NIR Spectrophotometer (Shimadzu UV-3101(PC), Japan). BaSO_4_ was used as the reference baseline for the absorption and reflection measurement. The spectrum wavelength was in the range of 200–800 nm. The reflection value of each sample was determined for the energy band gap by following the Kubelka–Munk method combined with the Tauc relation^33,34^:

5 $$\left( {\upalpha {\text{h}}\upnu } \right) = {\text{A}}({\text{h}}\upnu - {\text{E}}_{{\text{g}}} )^{{\text{n}}}$$

where α is absorption, h is the Plank's constant (6.626 × 10^–34^ J s), ν is the frequency of photons, A is a proportionality constant, E_g_ is the average band gap of the material and n = 1/2 for indirect transmission. The indirect average bandgap transition energies were estimated from the intercepts of the linear portion of the (αhν)^1/2^ versus hν plots.

Photoluminescence spectra of all TiO_2_ samples were measured on a photoluminescence (Multichannel spectrometer, Avantes) with LED laser under excitation at 255 nm.

The change of chemical structure by photocatalytic reaction of RhB was determined by NMR technique. All samples for ^1^HNMR spectra were dried at 60 ℃ for 12 h and then dissolved in D_2_O before measured by NMR (400 MHz) (Bruker, Model: Ascend-400 (Prodigy unit)).

### Photocatalytic activity test

Photocatalytic activity of different crystalline TiO_2_ on cellulose support under UV and visible light irradiation on Rhodamin B (RhB) degradation was determined. In the typical procedure, the catalyst (1 g/L) was added to a 50 mL aqueous solution of RhB dye (5 ppm). The mixture was agitated at a speed of 250 rpm in the dark for a time interval of 30 min to reach the adsorption–desorption equilibrium. Afterward, the mixture was irradiated using a lamp (Philips LED 18W) with an intensity of 6900 lx for visible light, while UV irradiation by using a UV lamp (Blacklight T5 8W, wavelength 365 nm (UVA)) with an intensity of 0.180 mW/m^2^. Aliquots were collected from the photoreactor at a given time and then centrifuged to remove solid residues from the supernatant. The concentration of RhB in the supernatant was measured by UV–Vis spectrophotometer (Agilent Technologies, USA.). The degradation percentages, D % of the RhB dye were calculated using Eq. (6)^35^:

6 $${\text{D}}\left(\%\right)=\frac{{C}_{0}-C}{{C}_{0}} \times 100$$

where C_0_ is the initial concentration of the dye and C is the concentration at various times.

### Batch recycle

An experiment was conducted to determine the remaining activity of cellulose-TiO_2_ as a catalyst. The method was modified following that by Lin et al.^36^. The catalyst regeneration was carried out using the RhB solution volume based on the catalyst amount from the previous cycle. After the 1st cycle, the catalyst was separated by centrifuge at 5000 rpm, then washed with DI water and dried in an oven at 40 °C for 24 h. In the next cycle, the volume of RhB at 5 ppm was controlled by the weight of catalysts from the last cycle, and similar conditions were used for photodegradation.

## Results and discussion

### Effect of H_2_SO_4_ concentration on crystalline structure of TiO_2_ on cellulose

TiO_2_ on cellulose support was fabricated through an in situ hydrolysis process. Titanium oxysulfate is a precursor that can be dissolved in water and acidic conditions. Thus, the effect of H_2_SO_4_ concentration, ranging from 0 to 10% wt on the TiO_2_ phase, was investigated by setting TiOSO_4_ concentration, hydrolysis temperature, and time to be 0.25 M, 90 ℃, and 8 h, respectively.

**Figure 1:**
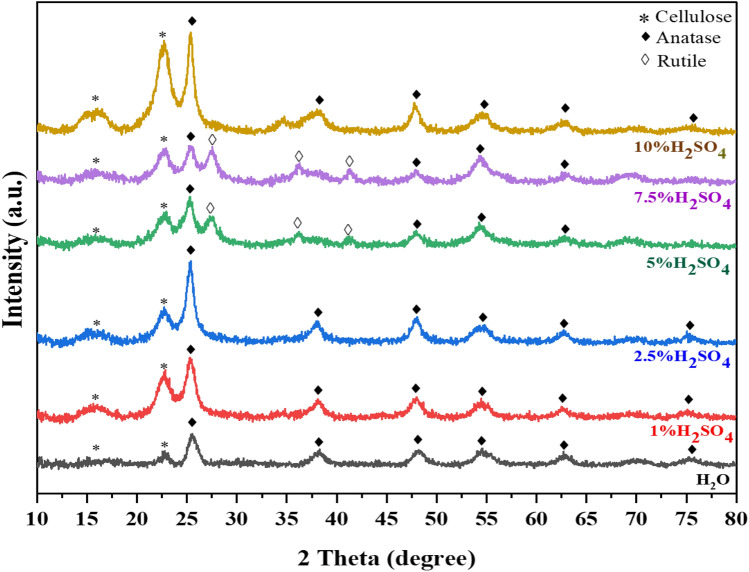
X-ray diffraction patterns of TiO_2_ on cellulose with different H_2_SO_4_ concentration at 90 ℃ for 8 h.

Figure 1 shows the XRD pattern of TiO_2_ on cellulose support at various H_2_SO_4_ concentrations. It is evident that the peaks corresponding to cellulose were observed in all samples at 2θ angles of 15.80° and 22.59°, indicating the presence of the (110) and (200) planes of cellulose type I^37^. The peak intensity at 2θ of 15.80° and 22.59° was increased with higher H_2_SO_4_ concentration. This suggested that the acidic condition not only preserved the crystalline structure of cellulose but also eliminated the amorphous regions. It was also observed that the peaks at 2θ of 25.30°, 38.11°, 47.96°, 54.54°, 62.61°, and 75.20° which represented (101), (004), (200), (105), (204), and (215) that referred to anatase phase TiO_2_ in all the samples, except for TiO_2_ on cellulose support at 5%wt and 7.5%wt of H_2_SO_4_. The new peaks were found at 2θ of 27.47°, 36.18°, and 41.20° which indicate (110), (101), and (111) of rutile phase. While at 10%wt of H_2_SO_4_, only the anatase phase of TiO_2_ was found. A possible explanation that an increase of H_2_SO_4_ concentration affected to the crystalline phase of TiO_2_ will be later discussed in the section which deals with the mechanism of TiO_2_ formation on cellulose. The hydroxyl groups on cellulose could enhance the nucleation and growth of TiO_2_ nanocrystals^7^. The crystalline size and percentage of anatase and rutile were calculated by Eqs. (2) and (4), with the results shown in Table 1. The percentage of rutile increased to nearly 10% when H_2_SO_4_ concentration was increased from 5 to 7.5%wt. The crystalline size of anatase and rutile were smaller in the range of 5.80–9.58 nm when compared with commercial TiO_2_, of which crystalline size is more than 20 nm. The difference was due to the synthesis process in this research was without calcination. This result was consistent with the result of Shandilya and Capron^20^. To study the effects of temperature and time on the phase of TiO_2_ from hydrolysis of TiOSO_4_ on cellulose, we selected 5%H_2_SO_4_ and 10% H_2_SO_4_ concentration in the experiment.

**Table 1:** The ratio and crystalline size of anatase and rutile at different H_2_SO_4_ concentration.

Conditions	Ratio (%)		Crystalline size(nm)	
	Anatase	Rutile	Anatase	Rutile
H_2_O	100		7.82	
1%H_2_SO_4_	100		8.7	
2.5%H_2_SO_4_	100		9.24	
5%H_2_SO_4_	58.82	41.18	6.88	6.81
7.5%H_2_SO_4_	48.43	51.57	6.46	7.43
10%H_2_SO_4_ or cellulose-anatase	100		9.58	

### Effect of temperature on the crystalline structure of TiO_2_ on cellulose

**Figure 2:**
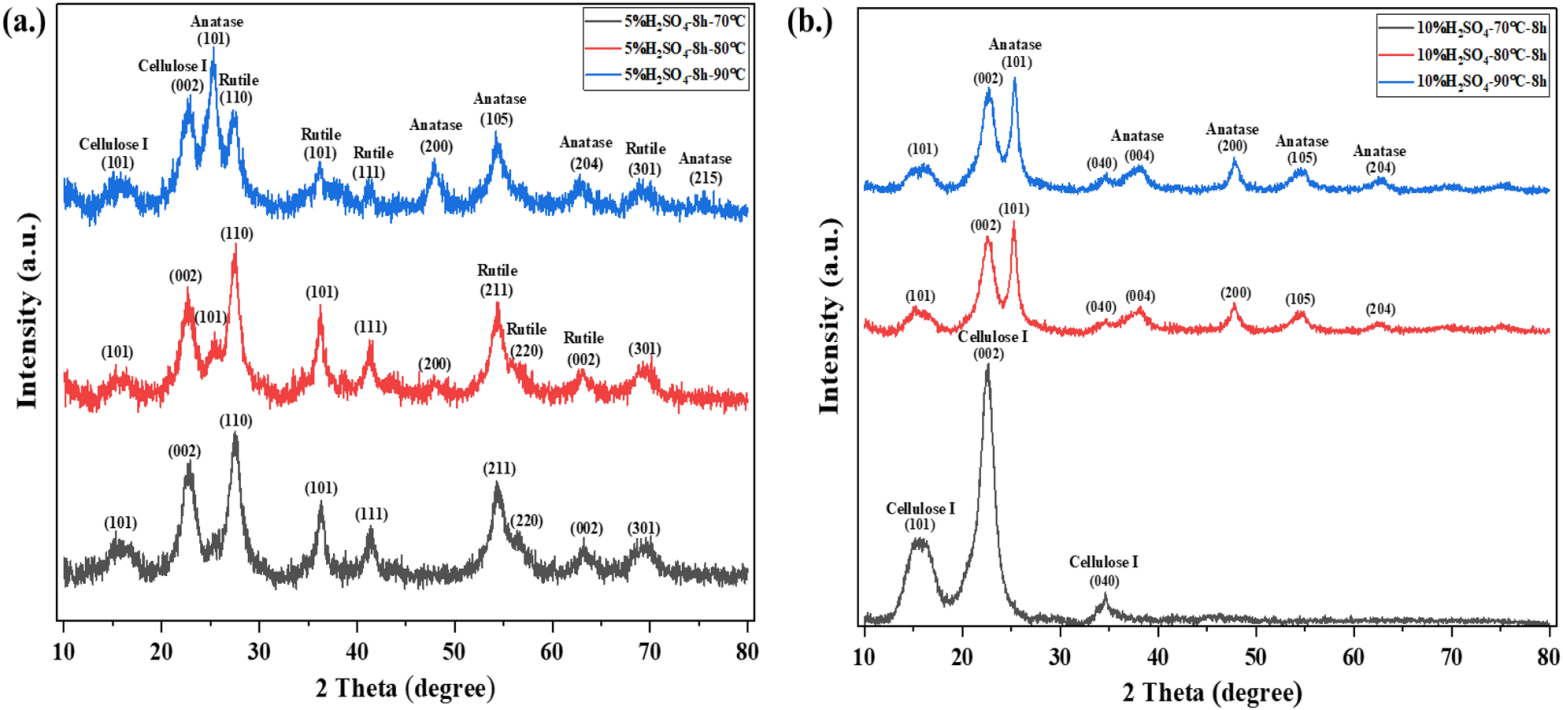
X-ray diffraction patterns of TiO_2_ on cellulose at 5%H_2_SO_4_ (**a**) and 10%H_2_SO_4_ (**b**) at different temperatures.

The effect of temperature on the crystalline phase of TiO_2_ on cellulose support at 5%H_2_SO_4_ and 10%H_2_SO_4_ is depicted in Figs. 2(a) and (b), respectively. In this study, the temperature was 70, 80, and 90 ℃ since it was desirable not to degrade the cellulose. The crystalline structure of cellulose was found to have similar peaks for all samples using 5%H_2_SO_4_ and 10%H_2_SO_4_ even though the temperature was varied from 70 to 90 ℃. On the contrary, the peak of TiO_2_ significantly changed when the temperature increased from 70 to 90 ℃ at 5%H_2_SO_4,_ as shown in Fig. 2(a). At 70 ℃, the peak appeared at 2θ of 27.57°, 36.32°, 41.63°, 54.49°, 56.72°, 63.41, and 69.58° which represent (110), (101), (111), (211), (220), (002) and (301) of the rutile phase^10^. Increasing the temperature to 80 ℃, a new small peak was detected at 2θ of 25.34° corresponding to (101) of the anatase phase. Finally, on increasing the temperature to 90 ℃, the peak at 25.34° was found to be higher than the peak at 27.40°. This result indicated that low acid concentration and low temperature were conducive to the formation of the rutile phase of TiO_2_. In another case, at a high acid concentration (10%H_2_SO_4_), the results are shown in Fig. 2(b). At low temperatures (70℃), no peak of crystalline TiO_2_ was detected due to the complete dissolution of TiOSO_4_ in 10%H_2_SO_4_^20^. In contrast, as evidenced in Fig. 2(b), increasing temperature to 80 and 90 ℃ caused the formation of crystalline anatase of TiO_2_ on the cellulose. The percentage and crystalline size of anatase and rutile were calculated and shown in Table 2. It should be noted that the anatase phase was generated in an amount of 5% when the temperature increased from 70 to 80 ℃. At 5%H_2_SO_4_, the crystalline sizes of anatase and rutile were similar and found to be ~ 5–6 nm even though the hydrolysis temperature was different. Consistent with the result of Fischer et al*.*^10^ and Björn Elgh & Anders E. C. Palmqvist^38^, the result indicated that acid concentration and temperature played an important role in the formation of crystalline TiO_2_.

**Table 2:** Ratio and crystalline size of anatase and rutile at different temperature.

Conditions	Ratio (%)		Crystalline size (nm)	
	Anatase	Rutile	Anatase	Rutile
5%H_2_SO_4_-70 ℃-8 h or cellulose-rutile		100		5.87
5%H_2_SO_4_-80 ℃-8 h	5.31	94.69	5.28	5.97
5%H_2_SO_4_-90 ℃-8 h or cellulose-mixed	58.82	41.18	5.8	5.68
10%H_2_SO_4_-70 ℃-8 h	N/A	N/A	N/A	N/A
10%H_2_SO_4_-80 ℃-8 h	100		10.54	

### Effect of Time on the crystalline structure of TiO_2_ on cellulose

**Figure 3:**
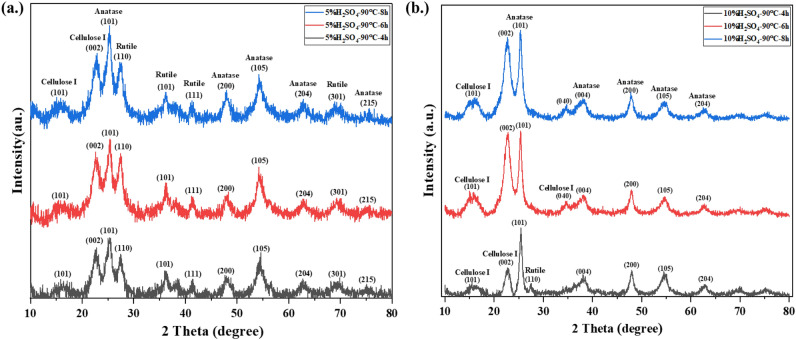
X-ray diffraction patterns of TiO_2_ on cellulose at different time 5%H_2_SO_4_ (**a**) and 10%H_2_SO_4_ (**b**).

The last parameter of interest in the synthesis of TiO_2_ in this work is reaction time. The reaction time was selected at 4, 6, and 8 h. The crystalline structure of TiO_2_ and cellulose are shown in Figs. 3(a) and (b) for 5%H_2_SO_4_ and 10%H_2_SO_4_, respectively at 90 ℃. Figure 3(a) exhibited the peaks corresponding to anatase and rutile phases. When synthesis time was increased, the peaks remained similar for all the synthesis times but the intensity of anatase and rutile slightly changed. The ratio of anatase and rutile was calculated and shown in Table 3. The percentage of rutile decreased from 56.02 to 41.18% when the reaction time increased from 4 to 8 h. In another condition at 10%H_2_SO_4_, and 90 ℃ the results are shown in Fig. 3(b). Similar and sharp peaks of anatase phase were found in all the samples regardless of reaction time. Only a small peak of rutile could be observed at 4 h.

**Table 3:** Ratio and crystalline size of anatase and rutile at different time.

Conditions	Ratio (%)		Crystalline size (nm)	
	Anatase	Rutile	Anatase	Rutile
5%H_2_SO_4_-90 ℃-4 h	43.98	56.02	4.7	6.09
5%H_2_SO_4_-90 ℃-6 h	55.55	44.45	5.52	4.59
10%H_2_SO_4_-90 ℃-4 h	84	16	12.2	N/A
10%H_2_SO_4_-90 ℃-6 h	100		10.34	

To study the properties and efficiency of different phases of TiO_2_ on cellulose, the samples obtained from various hydrolysis conditions, viz, 10% H_2_SO_4_, 90 ℃, 5% H_2_SO_4_ 90℃ and 5%H_2_SO_4_, 70 ℃ for 8 h called cellulose-anatase, cellulose-mixed and cellulose-rutile, respectively, were investigated. The crystalline index, crystalline size and d-spacing of all the samples were calculated as shown in Table 4.

**Table 4:** Crystalline size, crystallinity index of cellulose-TiO_2_ polymorphs.

Condition	Anatase		Rutile		Cellulose	
	Crystalline size (nm)	d-spacing (Å)	Crystalline size (nm)	d-spacing (Å)	CrI (%)	Crystalline size (nm)
Cellulose-anatase	9.58	3.51	–	–	88.46	5.31
Cellulose-mixed	5.80	3.53	5.68	3.25	78.02	5.03
Cellulose-rutile	–	–	5.87	3.24	74.72	5.17

### FTIR analysis

**Figure 4:**
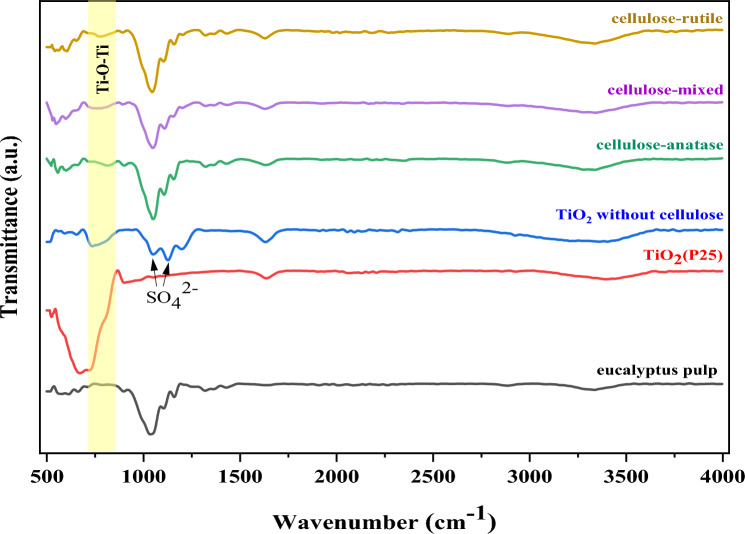
FTIR of TiO_2_ on cellulose.

The functional groups of all samples were analyzed by FTIR and the results are shown in Fig. 4. The spectra of eucalyptus pulp contain many peaks including broad peaks at 3330 cm^−1^, 2905 cm^−1^ and 1640 cm^−1^ which are related to O–H, C–H, and O–H stretching, respectively^39^. The sharp peak was exhibited at 1040 cm^−1^ which was associated with CO groups in the polysaccharide structure while the peaks at 1160 and 1104 cm^−1^ indicated C–O–C stretching^23^. These results confirmed that the eucalyptus pulp was cellulose type I^40^. The functional groups of TiO_2_(P25) exhibited peaks at 500–800 cm^−1^ referring to Ti–O bond^41^. After the synthesis of different crystalline structure of TiO_2_ on eucalyptus pulp, the spectra of cellulose exhibited similar peaks to those of eucalyptus pulp and TiO_2_(P25) but a small peak of TiO_2_ was observed on the samples due to low concentration of TiO_2_ on eucalyptus pulp. Moreover, a new broad peak appeared at 810 cm^−1^ which correspond to Ti–O–Ti that was generated from the hydrolysis of TiOSO_4_^42,43^. These results indicated that TiO_2_ from hydrolysis adhered to cellulose in the eucalyptus pulp. The peaks at 1052 and 1127 cm^−1^ were observed in TiO_2_ without cellulose which can be attributed to the characteristic of bidentate SO_4_^2−^ coordinated to metals such as Ti^4+^^44,45^.

### Raman spectroscopy

**Figure 5:**
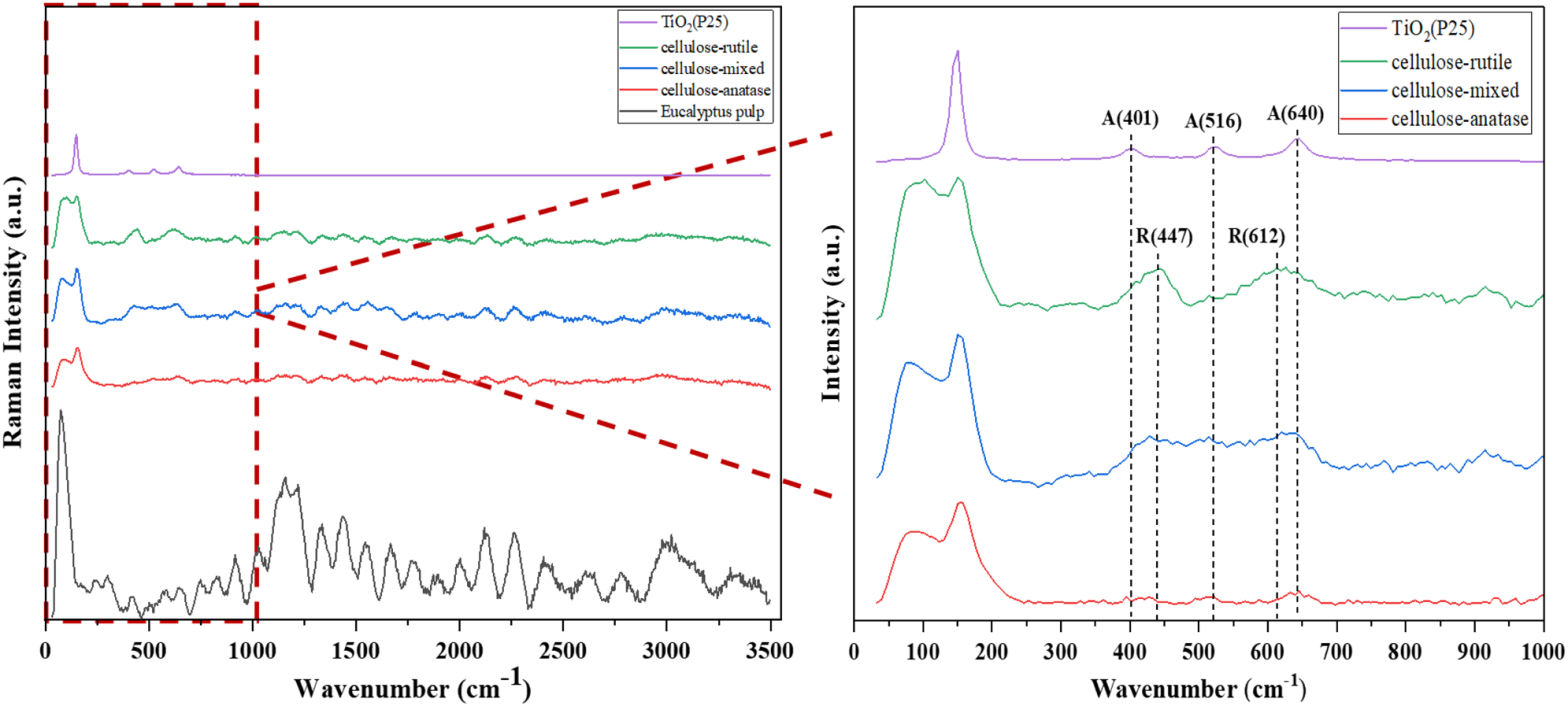
Raman spectroscopy of TiO_2_ on cellulose.

Since the FTIR spectra revealed only a small broad peak of TiO_2_, Raman spectroscopy was used to confirm the existence of TiO_2_ on the cellulose support. Moreover, the spectra in this technique can identify the crystalline phase of TiO_2_. The results of Raman spectroscopy are shown in Fig. 5. TiO_2_(P25) and eucalyptus pulp were used as control samples. The peaks of TiO_2_ can be observed in the range of 100–1000 cm^−1^. A sharp peak at 150–154 cm^−1^ was detected in cellulose-anatase, cellulose-mixed, and cellulose-rutile due to the spectra of the anatase phase and rutile phase being near one another^46^. Other peaks of cellulose-anatase were detected at 516 and 640 cm^−1^ while cellulose-rutile at 447 and 612 cm^−1^^47^. Corresponding to the spectra of cellulose-mixed were observed broad peaks between 447 and 640 cm^−1^. The results confirmed that different polymorphs of TiO_2_ were formed on the cellulose by hydrolysis of TiOSO_4_.

### Mechanism for generating the crystalline structure of TiO_2_ on cellulose

**Figure 6:**
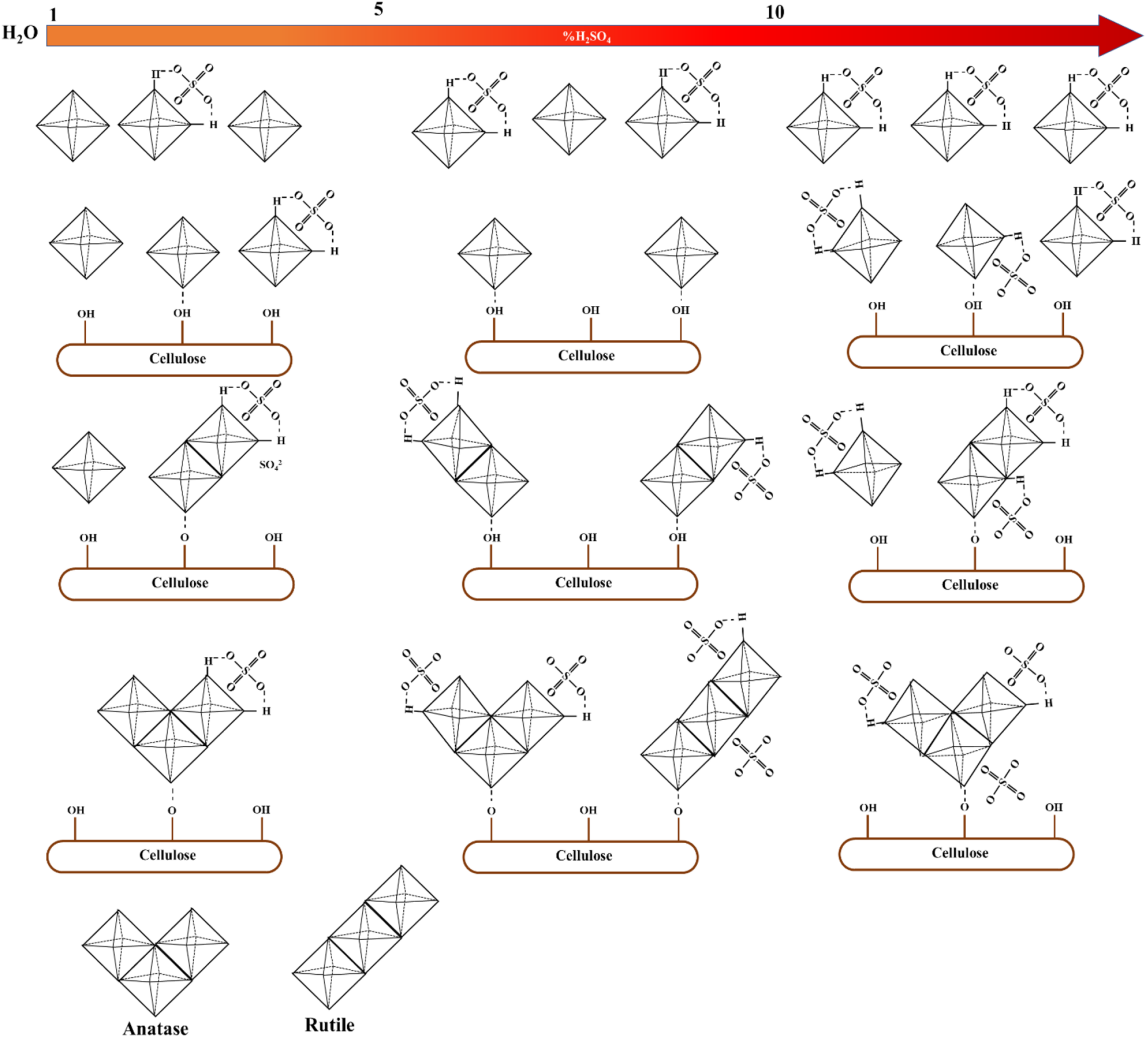
Proposed possible mechanism of the effect of H_2_SO_4_ concentration on the nucleation of TiO_2_ crystalline structure on cellulose.

As previously mentioned, the H_2_SO_4_ concentration and temperature of the hydrolysis process significantly affect the generation of the crystalline TiO_2_ phase on cellulose. A possible mechanism for the generation of the crystalline structure of TiO_2_ on cellulose is shown in Fig. 6. TiOSO_4_ reacts with water and H_2_SO_4_ to form TiO(OH)_2_, the hydroxyl group on cellulose surface adsorbs TiO(OH)_2_. TiO(OH)_2_ nuclei then form by hydrolysis condensation, which subsequently grows into TiO_2_ nanocrystals according to the following equations (7) and (8) ^1^:

7 $${\text{TiOSO}}_{{4}} + {\text{ 2H}}_{{2}} {\text{O }} + {\text{ H}}_{{2}} {\text{SO}}_{{4}} \leftrightarrow {\text{ TiO}}\left( {{\text{OH}}} \right)_{{2}} + {\text{2SO}}_{{4}}^{{{2} - }} + {\text{ 4H}}^{ + }$$

8 $${\text{TiO}}\left( {{\text{OH}}} \right)_{{2}} \leftrightarrow {\text{ TiO}}_{{2}} + {\text{ H}}_{{2}} {\text{O}}$$

Moreover, from Eq. (7) it can be seen that sulfuric acid will inhibit the formation of Ti–OH bonds, causing to slow down the hydrolysis reaction. Moreover, the heating rate in the dehydrating process could be a key factor in the arrangement of the TiO_2_ crystalline.

### TEM analysis

**Figure 7:**
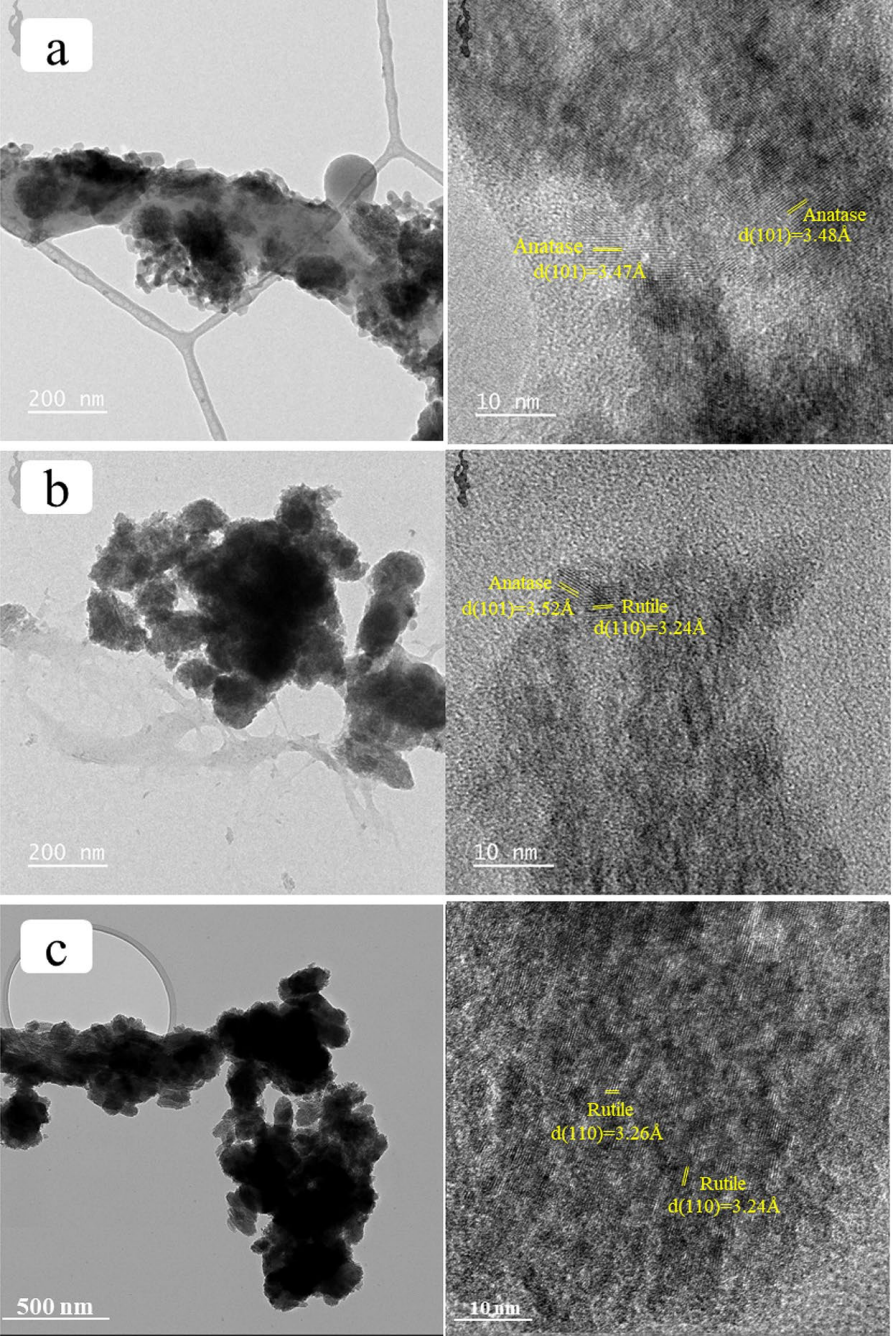
TEM images of cellulose-anatase (**a**), cellulose-mixed (**b**), and cellulose-rutile (**c**).

The structure and lattice spacing of TiO_2_ immobilized on cellulose were characterized by TEM and HRTEM, respectively, as shown in Figs. 7(a), (b), and (c). As seen in the TEM images of all samples, the shape of cellulose was found in a rod-like shape and was observed to decrease in size due to the destruction of cellulose by sulfuric acid. Moreover, due to the drying process, agglomeration of the particles was observed^5^. TiO_2_ particles appeared on the surface of the cellulose as black spots on the surface as shown in Figs. 7(a)–(c)^13^. The different phases of TiO_2_ exhibited different distributions and densities. The well-distributed anatase of TiO_2_ is shown in Fig. 7(a), while the clusters with higher density of mixed and rutile phases of TiO_2_ were observed in Figs. 7(b) and (c), respectively. Nevertheless, these results confirmed that the TiO_2_ was formed on the cellulose. The hydroxyl groups on cellulose were reported to be responsible for the nucleation of TiO_2_ crystals^4^. The size of TiO_2_ nanoparticles was found to be small and difficult to calculate for individual particles. Moreover, the particles tend to combine with neighboring particles by Ostwald ripening and grow into larger particles. This type of combination causes the mesoporous structure of TiO_2_ on cellulose^1^.

The HR-TEM was used to determine the lattice spacing for the identification of the crystalline phase of TiO_2_. The lattice fringes with spacings of 3.47–3.48 Å in Fig. 7(a) can be ascribed to (101) plane of TiO_2_ corresponding to the fringe distance of the anatase phase (3.46–3.52 Å)^48^. The lattice fringes with spacings of 3.24–3.26 Å in Fig. 7(c), which can be ascribed to the (110) plane, belonged to the rutile phase^49^. The lattice fringes with spacings 3.24 and 3.52 Å in Fig. 7(b) which are close to the (110) and (101) planes of rutile and anatase, respectively. These calculated results are consistent with the calculation of d-spacing from XRD results.

### FE-SEM analysis

**Figure 8:**
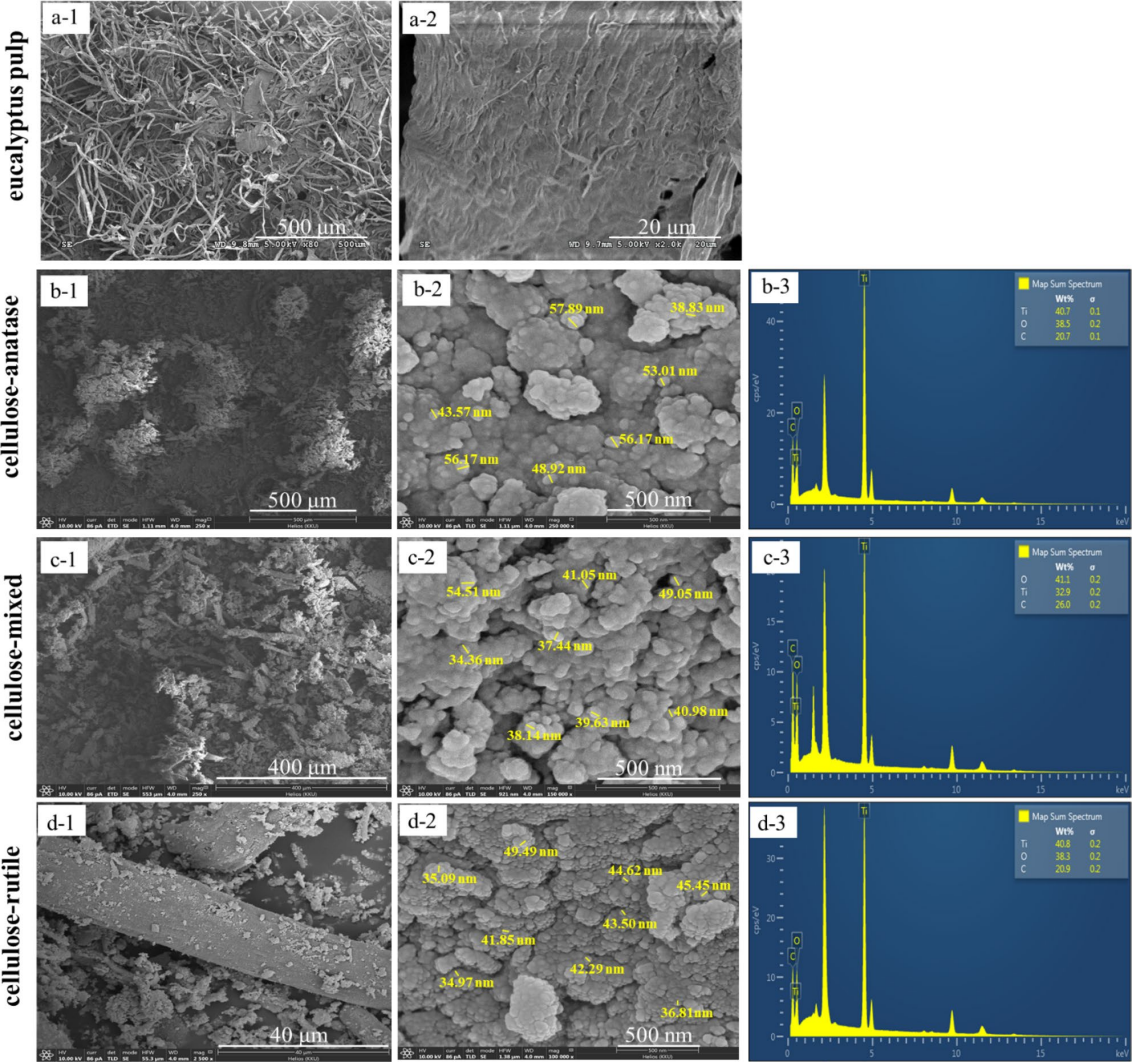
FESEM analysis of eucalyptus pulp (**a**), cellulose-anatase (**b**), cellulose-mixed (**c**) and cellulose-rutile (**d**).

Morphology of eucalyptus pulp before and after immobilization of TiO_2_ was observed by FESEM analysis, as shown in Figs. 8(a), (b), (c) and (d). Figure 8(a) is ascribed to the shape and surface of pure cellulose (eucalyptus pulp) before TiO_2_ formation. The shape of cellulose was long chain fiber while the surface was rough. Figures 8(b), (c) and (d) show the shape and surface of cellulose after being doped with different TiO_2_ crystalline phases from hydrolysis. On comparing the shape of cellulose, it was found that cellulose-anatase tended to be shorter than cellulose-mixed and cellulose-rutile because the cellulose-anatase was obtained by using high acid concentration (10%H_2_SO_4_), which caused destruction of the structure of cellulose by H^+^ from the H_2_SO_4_.

Similar shapes and particle sizes of different TiO_2_ polymorphs on cellulose are shown in Figs. 8(b2), (c2) and (d2). It is seen from these figures that small spherical particles of TiO_2_ fully covered the surface of cellulose. The average particle sizes of TiO_2_, which were calculated by image J program, were 47.24 ± 7.2, 41.82 ± 6.99 and 41.90 ± 5.16 nm for cellulose-anatase, cellulose-mixed and cellulose-rutile, respectively. In addition, it can also be seen the adherence between the TiO_2_ particles causes the structure to be raspberry-like. As reported in other work, the hydroxyl group can promote the heterogeneous nucleation and growth of inorganic particles^4^. In our results, the hydroxyl groups on the cellulose surface acted as support for the formation of the primary nuclei of TiO_2_ and subsequent growth into small crystals. The smaller crystals tend to combine into larger particles via Ostwald ripening to decrease the free surface energy^1^.

### TGA analysis

**Figure 9:**
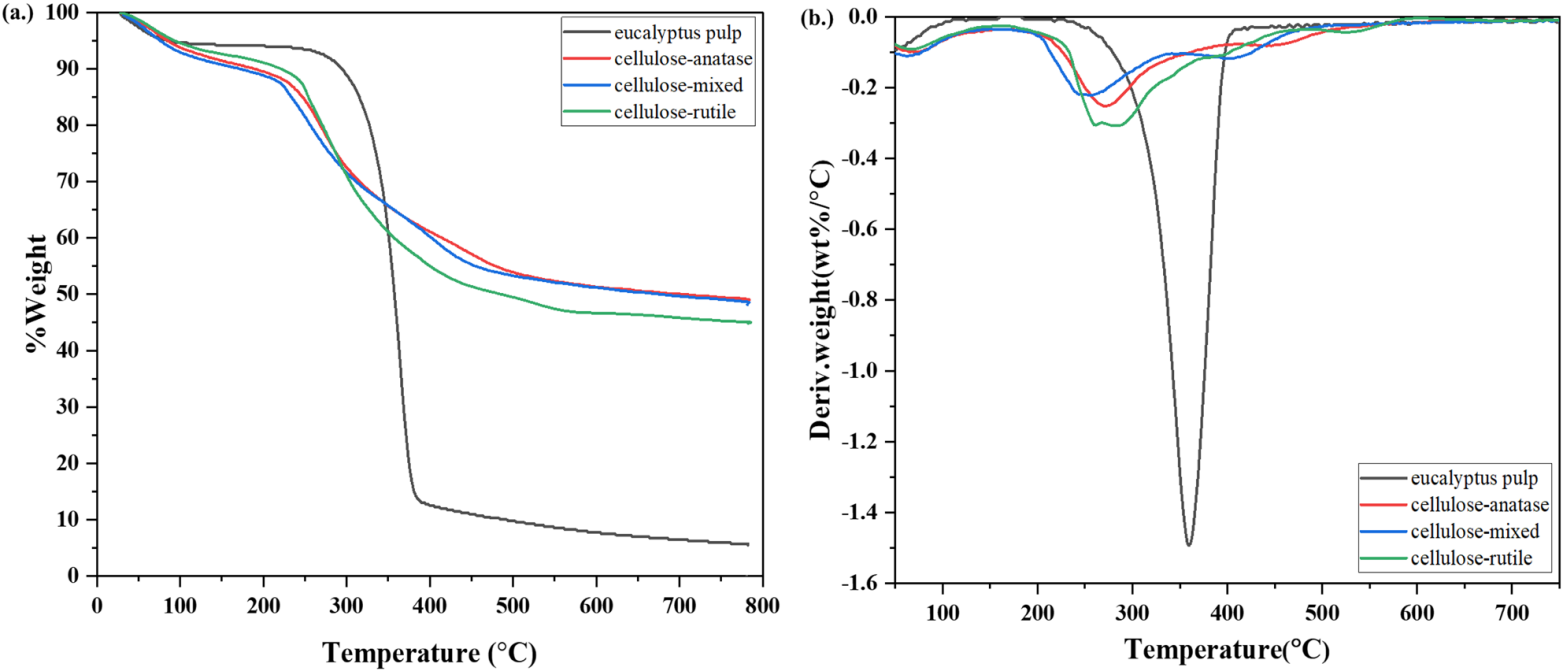
TGA (**a**) and DTG (**b**) analysis of eucalyptus pulp, cellulose-anatase, cellulose- mixed and cellulose-rutile.

Thermal stability and amounts of immobilized TiO_2_ nanoparticles on cellulose obtained from DTG analysis are shown in Fig. 9. Thermal degradation of eucalyptus pulp was detected in three stages. The first stage below 120 ℃ was attributed to the loss of water. High degradation was exhibited in the second stage which occurred between 250 and 380 ℃, normally referred to the decomposition of cellulose structure^13^. The final stage was the complete decomposition of cellulose. The degradation of cellulose-anatase, cellulose mixed and cellulose rutile had the same first stage but different in the second and third stages when compared with eucalyptus pulp. The decomposition took place between 200–350 ℃ and 350–500 ℃ for the second and the third stage, respectively. It should be noted that the second-stage decomposition temperature of cellulose was lower than that of eucalyptus. The lower temperature for the degradation of cellulose could be explained by the presence of H_2_SO_4_ in the system. The final weight from the TGA analysis indicated the amount of TiO_2_ generated from hydrolysis of TiOSO_4_ in percentage, numerically 36.35%, 33.13%, and 34.65% for cellulose-anatase, cellulose-mixed and cellulose-rutile, respectively. These results were comparable with those obtained from the EDS mapping of the percentage Ti element on the cellulose surface, as shown in FESEM images in Fig. 8.

### Photocatalytic activity

**Figure 10:**
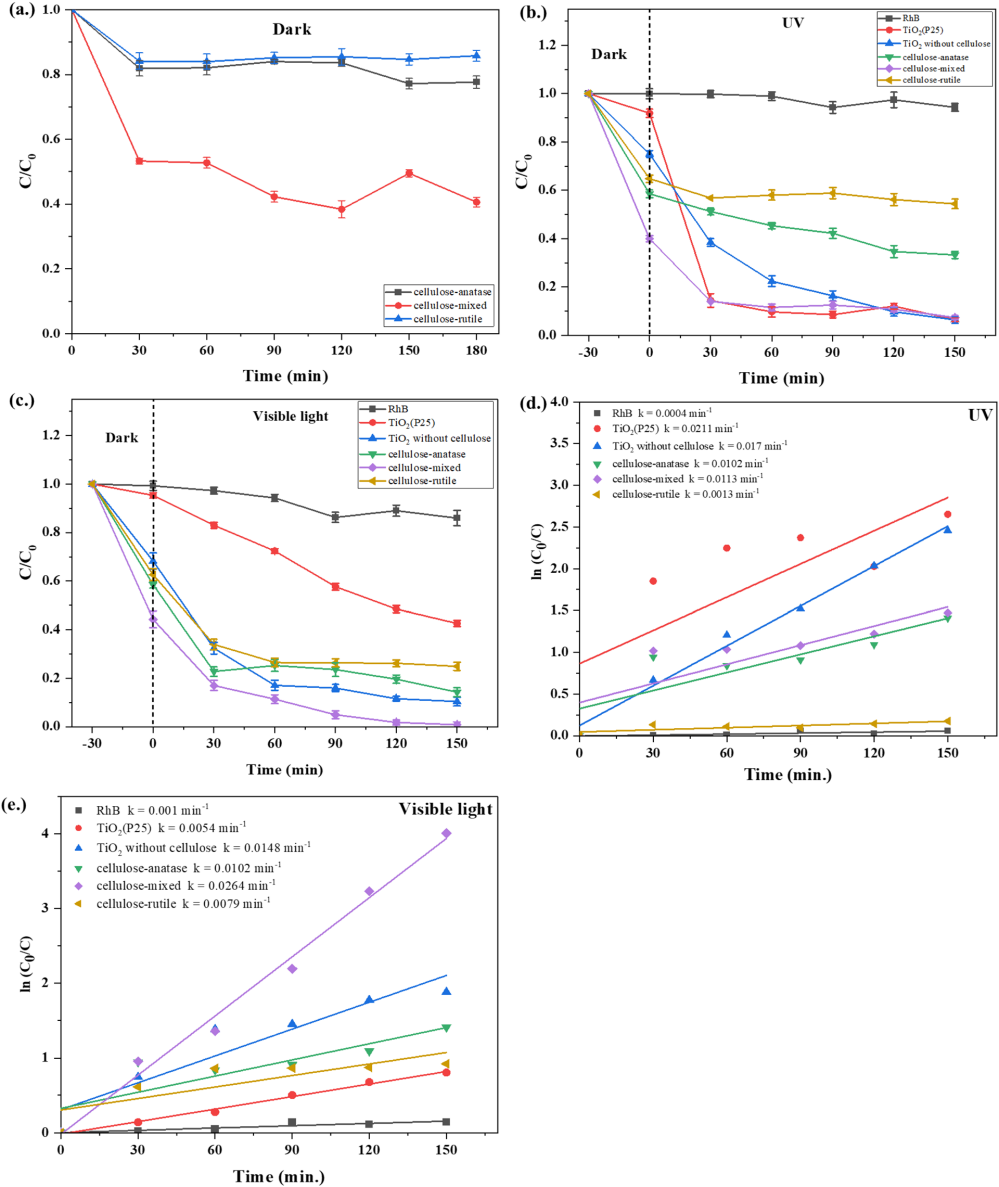
Degradation rate of RhB under (**a**) dark, (**b**) UV illumination, and (**c**) visible illumination, and the first-order kinetic plot of RhB under (**d**) UV illumination and (**e**) visible illumination.

In other previous studies, low-temperature synthesis of TiO_2_ on cellulose or nanocellulose by hydrolysis of TiOSO_4_ found only pure anatase and pure rutile phases of TiO_2_. The mixed phase of anatase and rutile can be obtained by calcination at high temperatures. In this study, we demonstrated a low-temperature synthesis to obtain mixed-phase TiO_2_ on cellulose. To compare the efficiency of different phases of TiO_2_ on cellulose in the photodegradation and kinetic reaction of rhodamine B, cellulose-anatase, cellulose mixed, and cellulose- rutile was tested under dark, UV illumination, and visible illumination conditions as shown in Figs. 10(a), (b), (c), (d) and (e), respectively.

Figure 10(a) illustrates the RhB adsorption of cellulose-anatase, cellulose-mixed, and cellulose-rutile after a duration of 180 min. The adsorption equilibrium was reached within the initial 30 min across all cellulose-TiO_2_ structures. The cellulose-mixed exhibited the highest adsorption capacity, reaching 50%, whereas the cellulose-anatase and cellulose-rutile displayed approximately 16% and 18% adsorption, respectively.

Figures 10(b) and (c) depict the RhB photodegradation reaction of RhB, TiO_2_(25), TiO_2_ without cellulose, cellulose-anatase, cellulose mixed, and cellulose-rutile under UV and visible light. The adsorption was first tested in the dark to reach an equilibrium state before the initiation of the photodegradation reaction. It was seen that cellulose-mixed adsorbed RhB around 56%, which was higher than cellulose-anatase (42%) and cellulose-rutile (38%), for both UV and visible light. The adsorption of RhB on the cellulose-TiO_2_ surface occurred by the electrostatic attraction between the negative charge from cellulose-TiO_2_ and the positive charge from RhB. This result agreed with that reported by Carneiro et al*.*^50^. They reported that the positive charge of RhB occurred at the ethyl group while the benzine ring exhibited the negative charge. Moreover, the shape of cellulose-mixed exhibited more voids between particles than cellulose-anatase and cellulose-rutile, thus causing higher adsorption of RhB by the cellulose mixed.

The self-degradation of RhB by UV and visible light irradiation was 6% and 14%, respectively. The RhB removal efficiency of cellulose-anatase, cellulose-mixed, and cellulose-rutile reached 66.73%, 92.63%, and 45.62%, respectively, after 150 min exposure to UV light, as shown in Fig. 10(b). For visible light in Fig. 10(c), the degradation efficiency of RhB was found to be 85.75%, 99.2%, and 75.08% for cellulose-anatase, cellulose-mixed, and cellulose-rutile, respectively. It was evident that the crystalline phase of TiO_2_ on the cellulose surface affected the efficiency of RhB degradation. The mixed phase between anatase and rutile exhibited better performance due to a lower energy band gap. Moreover, TiO_2_ without cellulose (1 g/l) was used to confirm that cellulose can improve the efficiency of TiO_2_. The degradation of RhB by TiO_2_ without cellulose revealed around 93.59 and 89.62% efficiency for UV and visible light, respectively. These results indicated that TiO_2_ on cellulose exhibited improved efficiency for RhB degradation in spite of the lower amount of TiO_2_ in the system. Comparisons between the cellulose-mixed and commercial TiO_2_(P25) under UV and visible light tests are shown in Figs. 10(b) and (c). The higher efficiency of cellulose-mixed than P25 was found under visible light, while both exhibited similar efficiency under UV light.

**Figure 11:**
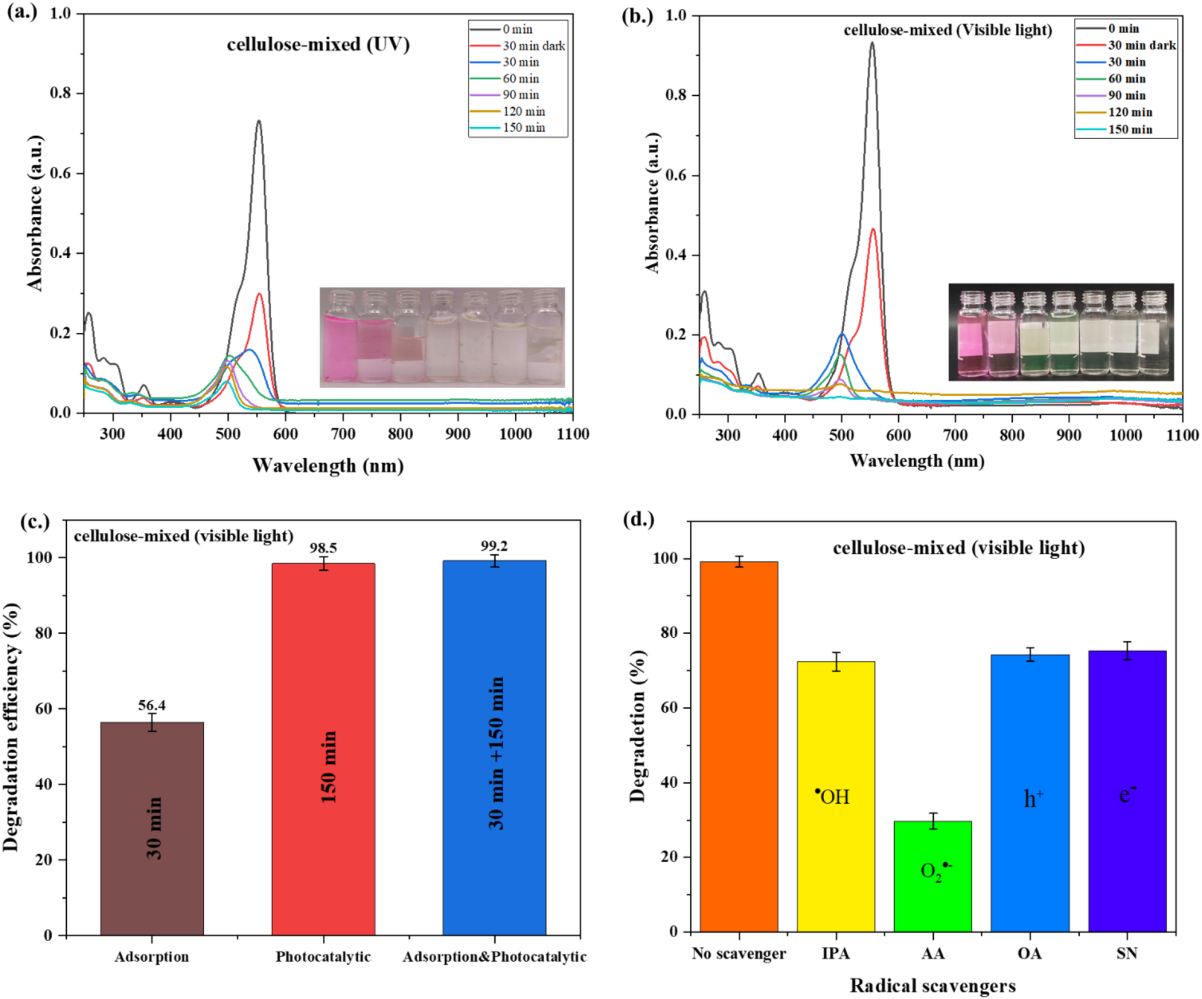
UV–Vis spectra of RhB solution as a function of (**a**) UV irradiation time and (**b**) visible irradiation time, (**c**) Degradation efficiency of RhB over cellulose-mixed under different processes and (**d**) radical scavengers on RhB photodegradation over the cellulose-mixed using visible light.

The first-order kinetics of RhB photodegradation under UV and visible light were computed and presented in Figs. 10(d) and (e), respectively. Additionally, the pseudo-first-order rate constant (k) was derived from the slope of the line. The results indicate that the rate constant (k) for the cellulose-mixed is twice as high as that of cellulose-anatase and TiO_2_ without cellulose in the degradation of RhB under visible light. Furthermore, Figs. 11(a) and (b) illustrate the UV–Vis spectrum of RhB after photodegradation by the cellulose-mixed structure under UV and visible light, respectively. The degradation efficiency of RhB under visible light with different processes and the impact of scavengers on RhB degradation by cellulose-mixed are presented in Figs. 11(c) and (d), respectively. Generally, the degradation of RhB under the photocatalytic process occurred by two pathways, namely cleavage of the whole conjugated chromophore and *N*-deethylation. In the cleavage of the chromophore, the main peak remained unchanged, but intensity decreased, while in the *N*-deethylation blueshifts of RhB peaks from 554 to 500 nm were detected by UV–Vis spectrometer^51^.

A rapid shift of peak was seen from 554 to 498 nm after being exposed to visible light irradiation for 30 min. then the peak intensity was observed to decrease, as shown in Fig. 11(b). Under UV light, the shift of peak occurred from 554 to 532, 502, and 498 nm after exposure to the light for 30, 60, and 90 min, respectively. After that, a decrease in peak intensity was observed, as depicted in Fig. 11(a). These results indicated higher activity of cellulose-mixed structure under visible light than UV light. Pictures of the product after irradiation are shown in Figs. 11(a) and (b). Figure 11(c) displays the degradation efficiency of RhB by cellulose-mixed under different processes, including adsorption, photocatalytic, and adsorption & photocatalytic reaction. The maximum RhB degradation efficiency of 99.2% was obtained by adsorption and photocatalytic. However, RhB degradation by photocatalytic was 98.5% dye removal. Thus, the RhB degradation efficiency in both cases was similar, indicating that the key factor of the RhB degradation with cellulose-mixed was more the electronic structure (mixed phase) of TiO_2_ than active adsorption sites.

The peak shifts explained the mechanism of RhB degradation when the cellulose-mixed was used as a catalyst. The first step was the adsorption of ethyl groups from RhB on the surface of the cellulose-mixed. After that, photocatalysis occurred by irradiation on the cellulose-mixed, and e^−^ from the valence band received more or equal energy to the energy bandgap; the e^−^ then went to the conduction band. At the valence band, h^+^ reacted with water to generate the hydroxyl radicals (OH^⋅^), while e^−^ at the conduction band reacted with oxygen to form superoxide (O_2_^⋅−^) according to the equations^52^;

$$\begin{aligned} {\text{Photoexcitation}}: & {\text{cellulose-mixed}}+ {\text{ h}}\upsilon \, \left( {{\text{visible}}} \right) \to {\text{e}}^{ - } + {\text{h}}^{ + } \\ {\text{Oxidation}}: & {\text{h}}^{ + } + {\text{H}}_{{2}} {\text{O}} \to {\text{OH}}^{ \cdot } + {\text{H}}^{{ + }{}} \\ {\text{Reduction}}: & {\text{O}}_{{2}} + {\text{e}}^{ - } \to {\text{O}}_{{2}}^{ \cdot - } \\ & {\text{RhB }} + {\text{ OH}}^{ \cdot } + {\text{O}}_{{2}}^{ \cdot - } \to {\text{photodegraded intermediates}} \to {\text{H}}_{{2}} {\text{O }} + {\text{CO}}_{{2}} \\ \end{aligned}$$

After the degradation of the ethyl groups, the cleavage of the benzine ring^53^ occurred until no peak of RhB was detected under UV–Vis, as shown in Figs. 11(a) and (b). This result confirmed that the complete degradation of RhB occurred when the cellulose-mixed was used as the photocatalyst.

Furthermore, a free radical scavenger test was conducted to explore the active species involved in RhB photodegradation. Isopropanol or propan-2-ol (IPA, 1:20 vol.) was introduced as the hydroxyl (^⋅^OH) radical scavenger^54^, ascorbic acid (AA, 2 mM) as the superoxide (^⋅^O^2−^) radical scavenger^55^, oxalic acid (OA, 25 mM) as the hole (h^+^)^56^, and silver nitrate (SN, 100 mM) as the electrons (e^−^)^57^.

The scavenging experiments for reactive oxygen species mirrored the photodegradation test. Prior to photodegradation, the reactive oxygen species were added to the RhB solution before introducing the catalyst. Figure 11(d) illustrates the impact of radical scavengers on RhB photodegradation over the cellulose mixed using visible light.

As depicted in Fig. 11(d), the absence of any scavenger resulted in 99% removal of RhB, while 75% degradation was observed with SN, 74% with OA, and 72% with IPA, respectively. About 30% of RhB degradation was observed with AA. These findings indicate that O_2_^⋅−^ plays a crucial role in RhB degradation, whereas ^⋅^OH, h^+^, and e^−^ have a relatively minor impact on the degradation process of RhB under visible light irradiation. Consequently, it can be inferred that superoxide is primarily responsible for the photodegradation of RhB.

### Energy bandgap

**Figure 12:**
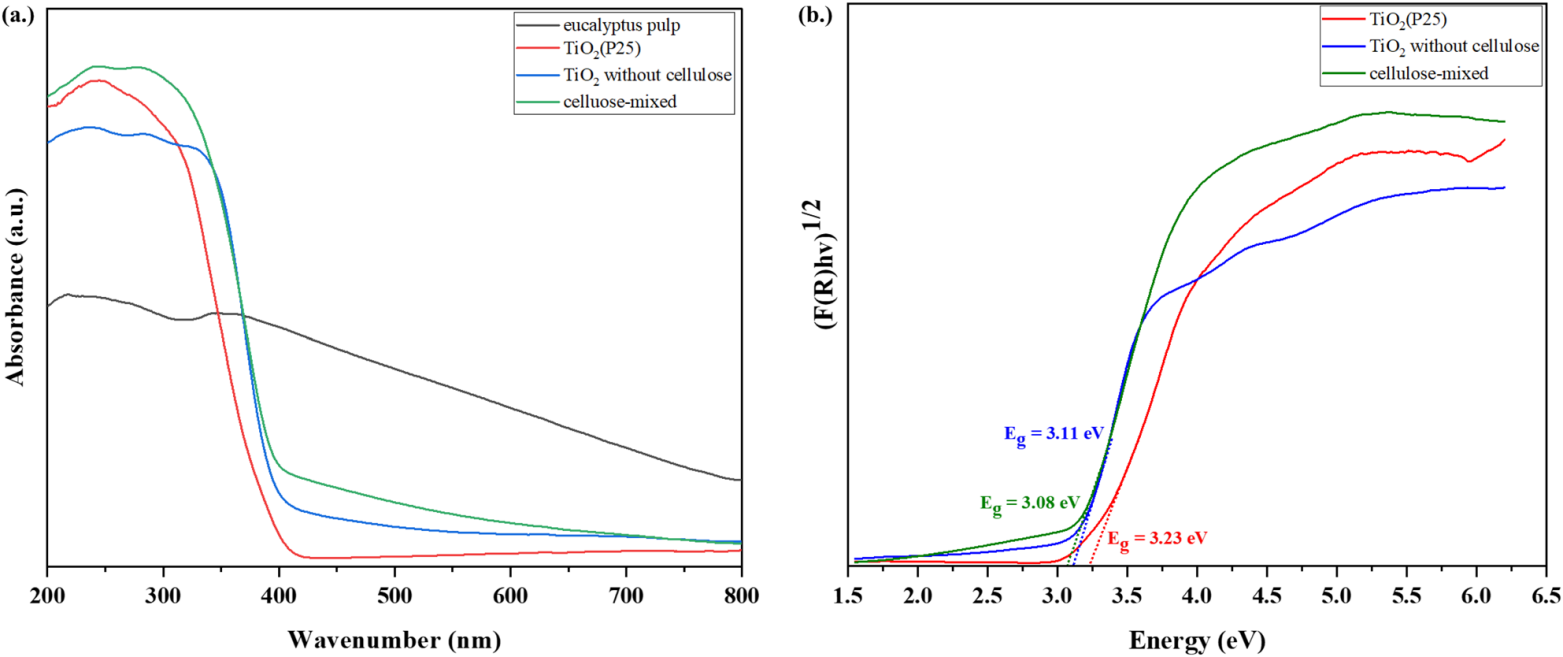
(**a**) UV–Vis DR spectra and (**b**) energy band gap values of eucalyptus pulp, TiO_2_(P25), TiO_2_ without cellulose and cellulose-mixed.

Optical properties of eucalyptus pulp, TiO_2_(P25), TiO_2_ without cellulose and cellulose-mixed were detected by UV–Vis DR, and the spectrum results are shown in Fig. 12 (a). It can be observed that the absorbance of the cellulose-mixed in the UV range between 200 and 400 nm was the highest as compared with commercial TiO_2_(P25) and synthesized TiO_2_ without cellulose. The stronger absorption in the visible light (400–800 nm) occurred in the eucalyptus pulp sample. As evidenced in Fig. 12(a), the absorption of radiation by TiO_2_(P25) over a narrow wavelength range indicated that TiO_2_(P25) was more active on UV rays than visible light. The higher absorbance of cellulose-mixed than that of the TiO_2_ without cellulose in the visible region indicated that the cellulose-mixed also performed better under visible light for photocatalytic application. The energy band gap was determined by plotting the Kubelka–Munk function against the proton energy, as shown in Fig. 12(b). The band gap value of cellulose-mixed was found to be 3.08 eV, smaller than TiO_2_ without cellulose (3.11 eV) and TiO_2_(P25) (3.23 eV)^58^. This result confirmed that cellulose could enhance the performance of TiO_2_ over a larger wavelength range, and the lower energy bandgap improved the photodegradation efficiency. Moreover, the performance of TiO_2_ over a greater wavelength range and lower energy band gap of the cellulose mixed can also improve the efficiency of RhB degradation under visible light.

### Photoluminescence analysis

**Figure 13:**
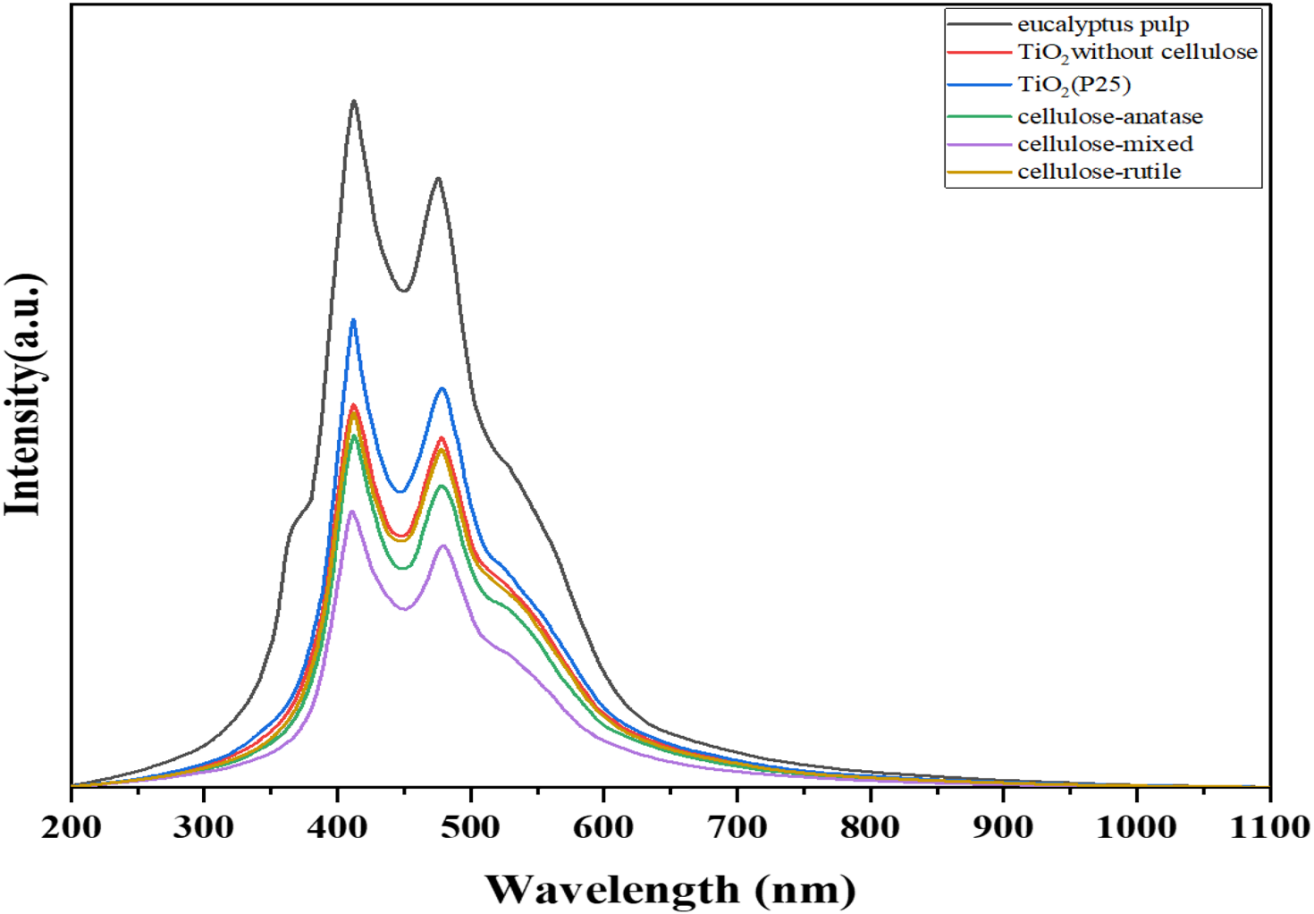
PL of eucalyptus pulp, TiO_2_ (P25), TiO_2_ without cellulose, cellulose-anatase, cellulose-mixed and cellulose-rutile.

It is well known that the energy band gap affects the photocatalytic efficiency. Moreover, the separation and transfer efficiency of photogenerated electron–hole pairs are the key factors of concern for photocatalytic activity^59^. The photoluminescence (PL) is a technique for probing the electronic structure, the transfer behavior of the photoexcited electron–hole pairs, and the rate of recombination of the material^60^. The recombination of photoexcited electron–hole pairs requires energy consumption, which increases the intensity of the spectrum, while the lower PL intensity indicates a higher separation rate of photoinduced electron–hole pairs. This could be the advantage of photocatalytic activity^61^. PL spectra of eucalyptus pulp, TiO_2_ without cellulose, TiO_2_(P25), cellulose-anatase, cellulose-mixed, and cellulose-rutile are shown in Fig. 13. The PL spectra of all samples exhibited emissions peaks at 411 and 475 nm, which can be described as the self-trapped excitons in TiO_6_ octahedral and the presence of oxygen vacancies in the TiO_2_ nanoparticles, respectively^62^. The peak of eucalyptus pulp corresponds to a report by Yi Ding Chai et al. that the broad PL spectra of cellulose appeared at 450–650 nm^63^. The unique emission behaviors of cellulose were thought to originate from the electron-rich oxygen and/or glucose units, which also confirmed the aggregation-induced or crystallization-induced emissions from cellulose^63,64^. It can be seen that all the samples showed similar peaks of PL while the intensity of PL spectra tended to decrease when TiO_2_ was immobilized on cellulose. The cellulose-mixed exhibited a lower PL intensity when compared with the cellulose-anatase and the cellulose-rutile. This result indicated that the cellulose-mixed had lower recombination of electron–hole pairs, which caused the electron and the hole to react with oxygen and water to produce highly reactive oxygen species that degraded pollutants via the oxidation processes^63,65^. The lower recombination of the cellulose-mixed could be due to the mixed phase anatase and rutile causing a lower energy band gap, hence a higher photocatalytic activity of RhB degradation.

### ^1^H NMR spectroscopy

**Figure 14:**
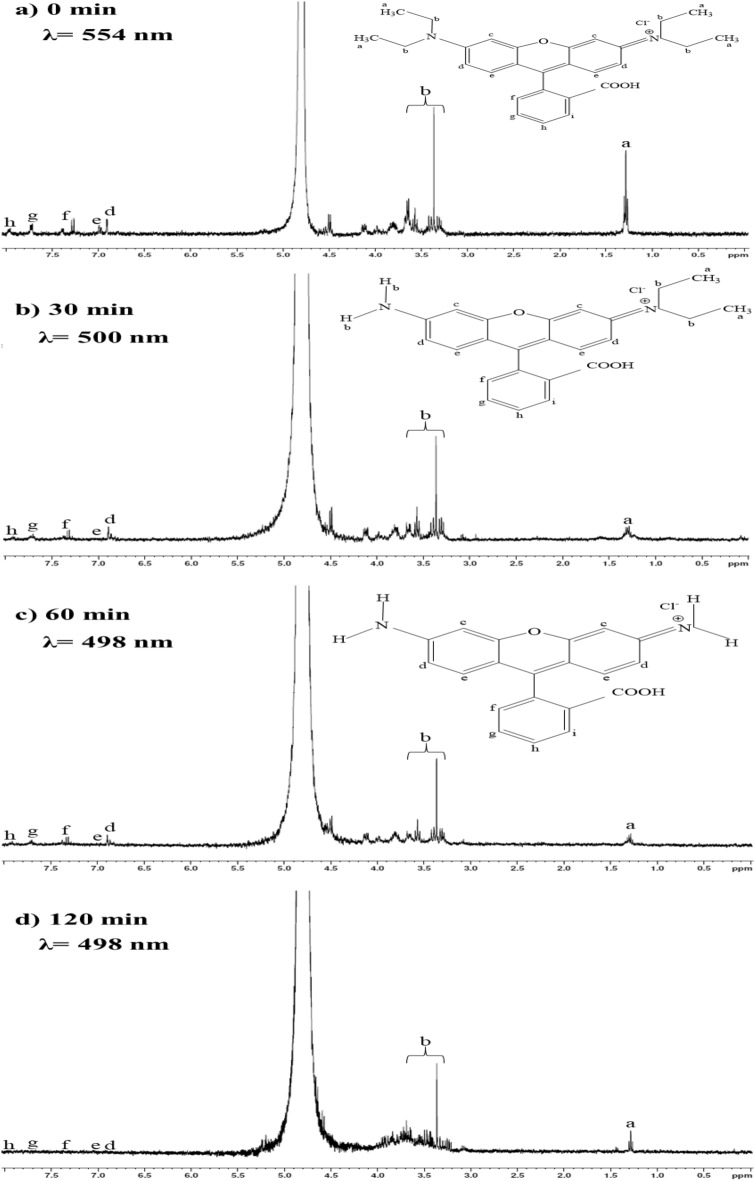
NMR spectrum of RhB at different degradation time.

To investigate the structure of RhB during the photocatalytic process, proton NMR analysis was performed. RhB solution after visible irradiation at 0, 30, 60, 90, and 120 min with the cellulose-mixed as catalyst was evaluated using ^1^H NMR, and the results are shown in Figs. 14 (a)-(d). The broad and sharp peak appeared at δ4.8 ppm, which indicated D_2_O solvent. In the beginning, the ^1^H NMR peaks of Ha and Hb of the ethyl groups were observed at δ1.30 (–CH_3_) and 3.30–3.65 ppm (–CH_2_–)^66^. The signal peaks of the aromatic hydrogen atoms Hd, He, Hf, Hg, Hh (d, e, f, g, and h referred to the H position in RhB structure) were exhibited at δ6.89, 6.95, 7.3, 7.7, and 7.9 ppm, respectively^67,68^. After 30 min irradiation, the peak of ethyl group at Ha was significantly decreased while that of Hb was slightly decreased. This result is consistent with the peak shift of UV–Vis from 554 to 498 nm to confirm the deethylation of RhB^69^. The decrease of aromatic hydrogen atom peaks was observed after irradiation of 60 min. After 120 min, no peak of the aromatic hydrogen atom was observed, indicating completion of RhB degradation. These NMR results indicated that the degradation of RhB under visible light by the cellulose-mixed occurred by the deethylation process at the initial stage of irradiation and then the breakup of chromophoric structure.

### Reusability

**Figure 15:**
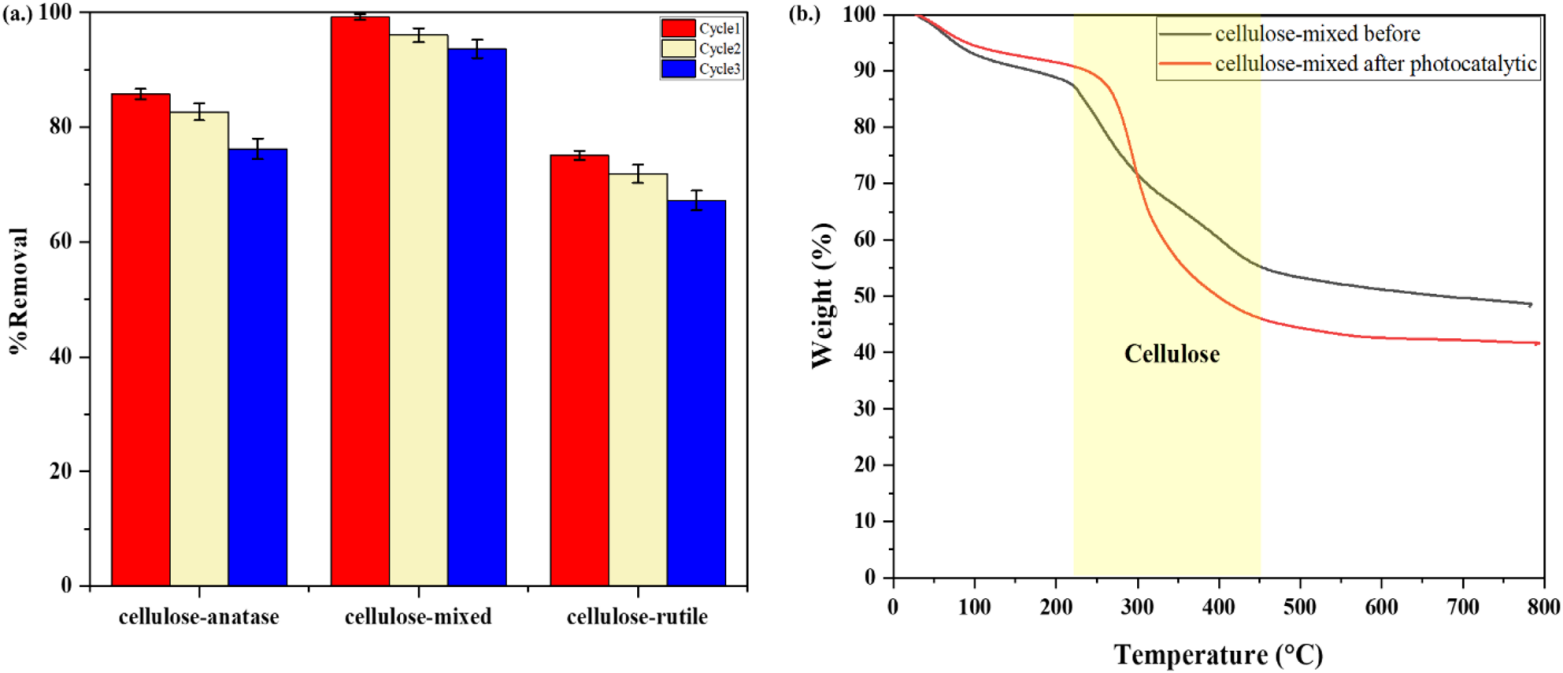
(**a**) Removal percentage of RhB by different catalysts under three cycles of visible light irradiation for 150 min and (**b**) TGA of cellulose-mixed before and after photocatalytic.

Good photocatalysis, stability, and reusability are important for continuous use in photocatalytic applications. To evaluate the recycling performance of the as-prepared catalyst, cellulose-anatase, cellulose-mixed, and cellulose-rutile were tested by RhB degradation for three cycles under visible light. After each run, the catalyst without desorption was used in the next cycle, and the results are shown in Fig. 15(a). The regeneration efficiency of the cellulose-mixed was found to be more than 90% for RhB degradation after three cycles, while the regeneration efficiencies were 75% and 65% for the cellulose-anatase and the cellulose-rutile, respectively. These results indicate that the cellulose-mixed exhibited good stability and reusability due to many active sites to enhance the photocatalytic activity.

To confirm the photostability of cellulose, TGA analysis was used to determine the weight of cellulose^70^ in the cellulose-mixed before and after photocatalytic reaction, as shown in Fig. 15(b). The result showed the weight of cellulose was increased from 45 to 55% due to the fact that cellulose could adsorb the residue RhB dye after 3 recycles of photocatalytic reaction. These results indicated that TiO_2_ did not affect cellulose degradation.

## Conclusions

The control of crystalline TiO_2_ polymorphs on cellulose was successful by hydrolysis of TiOSO_4_ at low temperatures. At lower H_2_SO_4_ concentrations (0–2.5%), the pure anatase phase was formed while an increase to 5–7.5% rutile phase occurred owing to the mixed phase of TiO_2_ and increasing to 10% the pure anatase was found. The pure rutile phase was formed at 70 ℃ while increasing temperature, and the anatase phase appeared. The formation of the pure anatase phase and mixed-phase depended on the H_2_SO_4_ concentration, while pure rutile was found at low temperatures. Cellulose acted as the template to direct the crystal growth of TiO_2_. The mixed phase of TiO_2_ on cellulose showed the highest photocatalytic activity (92.63% and 99.2% for UV and visible light, respectively) when compared with pure anatase and rutile phase for degradation of RhB after 150 min exposure. The degradation mechanism of RhB by using TiO_2_ on cellulose as a catalyst occurred by deethylation process. TiO_2_ on cellulose exhibited good stability and reusability. This study provides a simple method to generate the crystalline phase of TiO_2_, which exhibited a high potential catalyst for photodegradation without calcination in both UV and visible light systems. Additionally, it involves the customization of visible-light active cellulose-TiO_2_ nanocomposites with controlled crystalline structures to enhance photocatalytic performance.

## Data Availability

The datasets used and/or analyzed during the current study available from the corresponding author on reasonable request.
